# Transplantation of an allogeneic bone graft in treatment of post-sternotomy massive bone loss defects

**DOI:** 10.1186/1749-8090-10-S1-A130

**Published:** 2015-12-16

**Authors:** Martin Kaláb, Jan Karkoška, Milan Kamínek, Eva Matějková, Vladimír Lonský

**Affiliations:** 1 Department of Cardiosurgery, University Hospital Olomouc and Faculty of Medicine, Palacky University Olomouc, I. P. Pavlova 6, Olomouc, 775 20, Czech Republic; 2 National Cell and Tissue Centre, Palachovo náměstí 726/2, Brno, 625 00 Czech Republic; 3 Department of Nuclear Medicine, University Hospital Olomouc and Faculty of Medicine, Palacky University Olomouc, I. P. Pavlova 6, Olomouc, 775 20, Czech Republic

## Background/Introduction

Severe post-sternotomy dehiscence resulting in sternum and ribs losses, represent a surgical issue with mortality risk of 40%. Chest instability causes respiratory insufficiency, obstruction in disconnection from mechanical pulmonary ventilation and other soft tissue healing complications. Extensive bone tissue loss hinders the use of AO osteosynthesis.

## Aims/Objectives

Basing on orthopedic experience in bone defects replacement, we developed a technique of chest wall reconstruction using an allogeneic bone graft.

## Method

In the period 2011-2014, we performed allogeneic bone graft transplantation in 10 patients with extensive post-sternotomy defect of the chest wall. In 9 cases we used sternal graft, while in 1 case calva bone graft was used. Prior to the transplant, each patient underwent vacuum assisted closure treatment of the wound and general antibiotic therapy. Thorax and the graft stabilization was performed using transversal titanium plates with bicortical screws. In accordance with valid legislation, bone allograft was prepared by tissue centre. Powdered allogeneic spongy bone was used to enhance contact of graft and edges of sternal bone. In 9 cases the closure of soft tissues was performed using direct suture of mobilized pectoral flaps, while in 1 case V-Y transposition of pectoral flap was applied.

## Results

In 6 cases (60%) the reconstructed chest wall was successfully healed without further complications. In 3 cases (30%) additional resuture of soft tissues and skin in the wound lower part was performed during hospitalization. Nevertheless, a high stability of the chest wall with respiratory insufficiency improvement and a very good final cosmetic effect of the wound was achieved in these cases. Healing wasn't sufficient in 1 case (10%). Average length of the follow-up process of all the patients was 14.1 months (1-36). In four cooperating patients a scintigraphy examination of the chest wall was performed and it proved high healing activity of the graft and spongy bone.

## Discussion/Conclusion

Based on our existing experience, the transplantation of allogeneic bone graft seems to represent a promising method of management of severe sternal dehiscence. The procedure is easy to apply, with favourable functional and cosmetic effect.

